# Structural connectivity profile of scans without evidence of dopaminergic deficit (SWEDD) patients compared to normal controls and Parkinson’s disease patients

**DOI:** 10.1186/s40064-016-3110-8

**Published:** 2016-08-26

**Authors:** Mansu Kim, Hyunjin Park

**Affiliations:** 1 Department of Electronic Electrical and Computer Engineering, Sungkyunkwan University, Suwon, Korea; 2 School of Electronic and Electrical Engineering, Sungkyunkwan University, Suwon, Korea; 3 Center for Neuroscience Imaging Research (CNIR), Institute for Basic Science, Suwon, Korea

**Keywords:** Scans without evidence of dopaminergic deficit, Diffusion tensor imaging, Tractography, Correlation with clinical score

## Abstract

**Background:**

In this study, we investigated the structural connectivity profile of patients with scans without evidence of dopaminergic deficit (SWEDD) compared with normal controls (NC) and patients with Parkinson’s disease (PD). An accurate understanding of SWEDD is important so that appropriate therapeutic options can be presented to patients.

**Methods:**

Diffusion magnetic resonance imaging of NC (n = 40), SWEDD (n = 40) and PD patients (n = 40) was obtained from a research database. Tractography, the process of obtaining fiber information was performed. Connectivity analysis was performed on 16 connections in the cortico-basal ganglia-thalamo-cortical circuit. Group-wise differences among NC, PD and SWEDD patients were quantified in terms of structural connectivity based on fiber density. Then, we investigated correlations with the clinical score using the Movement Disorder Society-Sponsored Unified Parkinson’s Disease Rating Scale (MDS-UPDRS). A support vector machine classifier and leave-one-out cross-validation were applied to separate the NC, SWEDD and PD groups.

**Results:**

Pallidum–putamen and sensorimotor cortex–putamen connections showed significant group-wise differences among NC, PD and SWEDD patients and correlated with the MDS-UPDRS score.

**Conclusions:**

Pallidum–putamen and sensorimotor cortex–putamen connections might form a structural connectivity profile unique to SWEDD and could be a potential imaging biomarker for future movement disorder research.

## Background

Parkinson’s disease (PD) is a common neurodegenerative disorder characterized by motor symptoms and cognitive impairment (Stoessl 2011). Diagnosis of PD is typically performed according to the criteria from the United Kingdom’s Parkinson’s Disease Society Brain Bank. PD is associated with a loss of dopaminergic neurons in the substantia nigra (SN) that project to the striatum (Obeso et al. 2008). This dopamine imbalance causes inhibition of basal ganglia output and dysfunction within cortico-basal ganglia-thalamo-cortical circuits (CBGT) (Obeso et al. 2008; Sharman et al. 2013).

Functional neuroimaging techniques such as ^18^F dopa positron emission tomography or dopamine transporter single-photon emission computed tomography (DaT-SPECT) are adopted to assess dopaminergic dysfunction in PD patients. PD patients showed significantly reduced striatal uptake compared with normal controls (NC) using SPECT (Tolosa et al. 2006). However, approximately 10 % of clinically diagnosed PD patients have normal dopaminergic functional imaging and classified as having scans without evidence of dopaminergic deficit (SWEDD) (Schneider et al. 2007). To date, no consensus regarding the etiology of SWEDD exists. Some researchers consider it an early phase of PD, while others argue that it is very different from PD (Batla et al. 2014). Recent studies showed that abnormalities in cortical plasticity differed between PD and SWEDD patients (Schwingenschuh et al. 2010). Therapeutic options differ between SWEDD and PD because SWEDD patients are relatively insensitive to levodopa therapy (Fahn and Group 2005). Correctly understanding SWEDD is important so that appropriate therapeutic options can be presented to patients.

Diffusion tensor imaging (DTI) is a tensor based model of diffusion weighted magnetic resonance imaging (MRI) technique that can provide in vivo information on the microstructural integrity of brain tissue using anisotropic water diffusion. DTI data are processed with an algorithm known as tractography to perform the reconstruction of large white matter tracts. The processed fiber information is analyzed using connectivity analysis, which considers the brain as a complex network. Connectivity derived from DTI is known as structural connectivity. Various MRI techniques including resting state functional MRI (rs-fMRI) and DTI were applied to compare PD patients and NC (Kim et al. 2013; Sharman et al. 2013; Shu et al. 2011; Wu et al. 2009; Yu et al. 2013; Zhang et al. 2015; Ziegler et al. 2014). These studies reported PD related brain alterations compared with NC using rs-fMRI, DTI and track-based spatial statistics (Sharman et al. 2013; Zhang et al. 2015; Kim et al. 2013). A recent study investigated PD and SWEDD patients using structural connectivity and found four structural connections to explain a clinical score (Kim and Park 2016). That study explored the whole brain regions of interest (ROIs), while our study focused on regions of CBGT. The previous study adopted number of fibers but this study adopted a more refined measure of fiber density. The prior study considered PD and SWEDD, while this study considered three groups (i.e., PD, SWEDD and NC) to better characterize group differences.

In this study, the main objective was to investigate structural connectivity profile of SWEDD compared with NC and PD. A secondary objective was to investigate correlation between structural connectivity results with clinical scores of Movement Disorder Society-Sponsored Unified Parkinson’s Disease Rating Scale (MDS-UPDRS). We obtained diffusion MRI data from a research database, the Parkinson’s Progression Markers Initiative (PPMI) (Marek et al. 2011). Connectivity analysis was applied to brain regions of CBGT circuitry (Obeso et al. 2000, 2008). Group-wise differences among SWEDD, NC and PD patients were assessed in terms of structural connectivity based on fiber density. Significant connectivity differences were identified and further investigated with the MDS-UPDRS scores. Briefly, we identified structural connectivity profiles unique to SWEDD patients and found the identified connections were significantly correlated with MDS-UPDRS scores.

## Methods

### Subjects

This study was a retrospective analysis of anonymized imaging data and was approved by the Institutional Review Board (IRB) of Sungkyunkwan University. Our study did not require participant’s consent as we analyzed anonymized data. Participant data were anonymized and de-identified prior to analysis. The study included 120 participants classified into NC (n = 40), SWEDD (n = 40) and PD (n = 40) categories. The sub-groups were classified based on the criteria established by the PPMI consortium (Marek et al. 2011). Detailed criteria for the SWEDD group follows. First, the patients must have at least two of the following symptoms: (1) resting tremor, (2) bradykinesia, (3) rigidity (must have either resting tremor or bradykinesia). Patients were also included if they had asymmetric tremor or asymmetric bradykinesia. Second, patients were diagnosed of PD for 2 years or less at the time of screening with confirmation for no evidence of dopamine transporter deficit using DaT-SPECT imaging. Third, patients were not expected to require PD medication within at least 6 months from baseline. Fourth, patients were 30 years or older at the time of PD diagnosis. The age and sex ratios of each group were matched as shown in the Table 1. Details regarding the subjects, including MDS-UPDRS scores, are also shown in Table 1.

**Table 1:** Participant information

	NC	SWEDD	PD	p value (NC-SWEDD/SWEDD-PD/NC-PD)
Number of subjects	*n* = 40	*n* = 40	*n* = 40	–
Age (years) (mean ± SD)	60.92 ± 10.59	60.73 ± 10.72	61.86 ± 8.81	0.94/0.61/0.67
Sex (male/female)	24/16	26/14	26 /14	0.65/1/0.65
Disease duration (month) (mean ± SD)	–	6.41 ± 8.03	7.37 ± 7.79	<0.05/0.48/<0.05
MDS-UPDRS scores (mean ± SD)	0.57 ± 1.37	13.15 ± 9.17	22.67 ± 9.17	<0.05/<0.05/<0.05

Values are reported as the mean ± standard deviation (SD)

### Imaging data

We obtained diffusion-weighted and T1- and T2-weighted MRI data from the PPMI database (Marek et al. 2011). Diffusion images were acquired with a Siemens 3T scanner using the following parameters: 3T scanner, b = 1000 s/mm^2^, 64 diffusion gradient directions with 1 b0 image, image matrix = 116 × 116 × 72 and voxel resolution = 1.98 × 1.98 × 2 mm^3^. The subjects underwent T1- and T2-weighted MRI as well as image pre-processing steps required in addition to DTI data acquisition. Acquisition parameters for the T1-weighted images were as follows: TR = 2300 ms, TE = 2.98 ms, TI = 900 ms, image matrix = 240 × 256 × 176 and voxel resolution = 1 × 1 × 1 mm^3^. The parameters for the T2-weighted images were: TR = 3000 ms, TE = 101 ms, image matrix = 228 × 256 × 48 and voxel resolution = 0.9375 × 0.9375 × 3 mm^3^.

### Image pre-processing

**Fig. 1:**
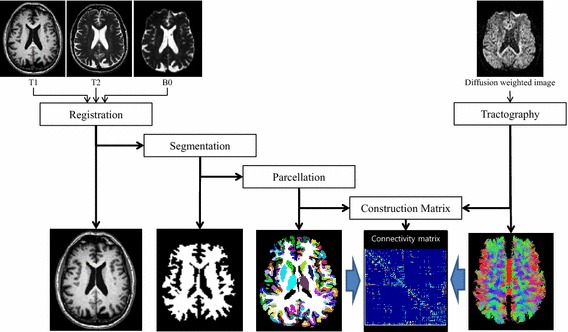
Image pre-processing procedures

Pre-processing steps were required to extract fiber information from the DTI data. Excellent review articles on this procedure exist, and thus, only a brief summary is given below (Daducci et al. 2012). Image pre-processing was performed using the Connectome Mapping Toolkit (CMTK), a Python-based open-source software (www.cmtk.org) (Daducci et al. 2012). For each subject, distortions caused by eddy currents and simple head motion during scans were corrected using FSL’s Eddy current tool and MCFLIRT (Smith et al. 2004). Then, the T1-, T2- and diffusion-weighted images were aligned to the Montreal Neurological Institute space with non-linear registration using FSL (Smith et al. 2004). The registered T1-weighted image was segmented into white matter, grey matter and cerebrospinal fluid using Freesurfer (Fischl 2012). The segmented white matter was later used to guide the tractography algorithm. The complete image pre-processing procedures are provided in Fig. 1.

### ROI specifications

**Fig. 2:**
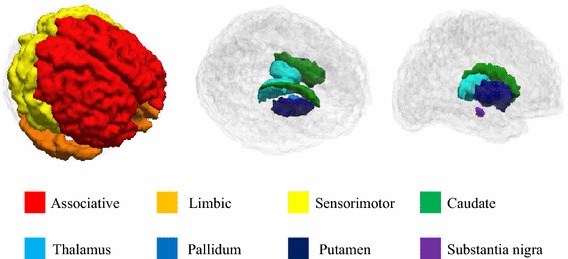
ROI specifications. A total of eight ROIs were defined from T1 and T2-weighted images, including the associative, limbic, sensorimotor cortex, caudate, putamen, pallidum, thalamus and substantia nigra regions

Connectivity analysis requires that ROIs are specified so that correlations among them can be investigated. Our analysis focused on the CBGT circuit, which consisted of eight regions: caudate, putamen, pallidum, thalamus, SN, sensorimotor cortex, associative cortex and limbic cortex, as shown in Fig. 2. The sensorimotor circuit, which includes the pre-central, post-central and para-central gyrus (Brodmann areas 1–5), is related to motor symptoms in PD (Albin et al. 1989). The associative circuit, which includes the dorsolateral prefrontal, middle and superior frontal cortex (Brodmann areas 8, 9, 44–47), is concerned with executive function and is affected by age and PD (Leh et al. 2010). The limbic circuit, which includes the medial temporal, orbitofrontal, posterior and anterior cingulate cortex, insula, entorhinal, hippocampus and amygdala, is related to stuttering and other movement disorders (Purves et al. 2008). All eight regions are structurally connected to the striatum (Parent and Hazrati 1995). Subcortical structures, caudate, putamen, pallidum and thalamus were specified by segmentation results from the registered T1-weighted images using Freesurfer (Fischl 2012). The SN region was specified by transferring ROI information from a pre-defined atlas via image co-registration (Keuken et al. 2014). The co-registration mapped the atlas information onto the subject’s image space so that both images reside on the same spatial framework. Three cortical structures, the sensorimotor, associative and limbic regions, were specified by the Desikan–Killiany anatomical atlas (Desikan et al. 2006).

### White matter tractography

**Fig. 3:**
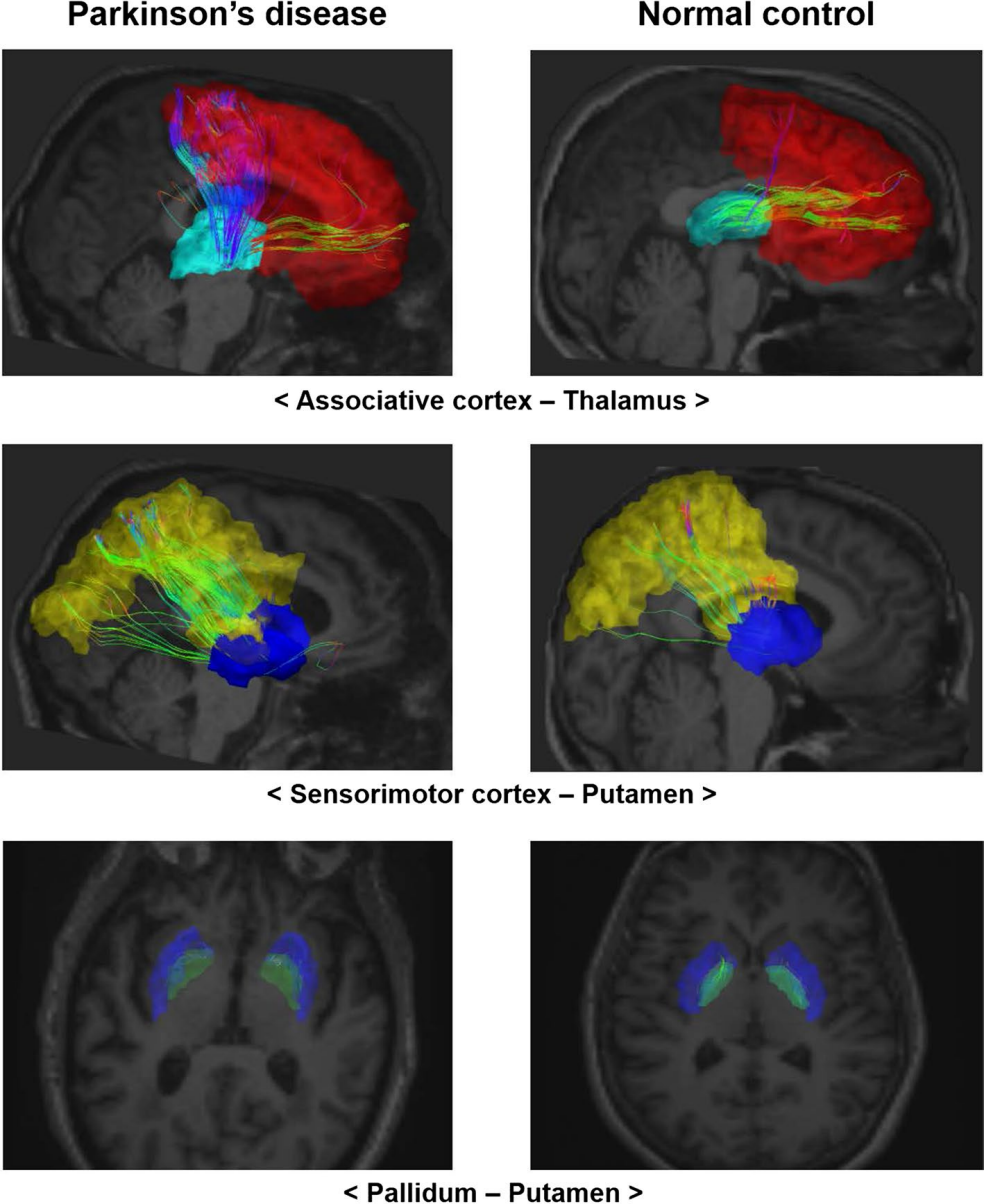
Illustration of tractography. The tracked fibers of three significant connections for representative PD patients and NC. PD cases are listed in the *first column* and NC cases are listed in the *second column*. *Colored regions* denote different ROIs. Fibers are displayed in streamlines. *Top sub-figures* associative cortex–thalamus connection. *Middle sub-figures* Sensorimotor cortex–putamen connection. *Bottom sub-figures* Pallidum–putamen connection

Fibers were constructed using tractography implemented using the Diffusion Toolkit (Wang et al. 2007). Fractional anisotropy (FA) of each voxel was computed using diffusion tensor data. Tractography was performed using the fiber assignment by continuous tracking (FACT) algorithm implemented in the Diffusion Toolkit to reconstruct all of the brain fibers (Wang et al. 2007; Mori and van Zijl 2002). The FACT algorithm propagated a line from the center of a seed voxel along the direction of the dominant vector, which was determined by the largest eigenvector of the tensor until the streamline exited to the next voxel. The starting point of the next voxel was the intercept of the previous voxel. Seed voxels were all voxels in the eight ROIs and connections were retained only if the seeds reached the one of the remaining ROIs. The tractography terminated when the algorithm entered a region with an abrupt change in fiber direction angle more than 60° and was limited to white matter regions and their neighbors, as fibers mainly exist in white matter. Illustrations of tracked fibers of the three significant connections for representative PD patients and NC are shown in Fig. 3.

### Structural network construction

Structural connectivity was assessed using 8 regions as nodes and 16 connections of interest as edges of a graph. There were 28 (=8 choose 2) possible connections between 8 regions and only 16 connections of the CBGT circuits were considered, as shown in the first column of Table 2. We applied a threshold of five fibers so that only structural connections with more than five tracked fibers connecting two regions were considered. This approach was chosen to reduce the chance of falsely identifying connections and thus leading to a more robust analysis (Shu et al. 2009, 2011; Im et al. 2014). The nodes were brain ROIs in the CBGT circuit, as described previously. Edge values were structural connectivity values between nodes. Structural connectivity values were defined as the fiber density, defined as the product of the connection density (CD) and the connection efficacy (CE) of the fibers (Hagmann et al. 2010). Our measure of CD incorporated the number of fibers connecting the regions and then normalized for the area of the brain regions and length of the fiber connecting two regions (Fischi-Gómez et al. 2015; Hagmann et al. 2008). The CD between two regions was defined as follows:

$$CD\left( {i,j} \right) = \frac{2}{{S_{i} + S_{j} }}\mathop \sum \limits_{f \in fibers}^{{}} \frac{1}{l\left( f \right)},$$

where f is the fiber connection between regions i and j, $l\left( {\text{f}} \right)$ is the length of the fiber connection and ${\text{S}}_{\text{i}}$ is the surface area of region i. Mean FA value along the fiber connections was used as the CE measure and FA is related to the fiber integrity. FA values reflect the degree of anisotropic water diffusion and is influenced by axonal myelination and the diameter and has a high correlation with conductivity (Tuch et al. 2001; Hagmann et al. 2010; Fischi-Gómez et al. 2015; Feldman et al. 2010). Many other studies also adopted FA to assess CE (Shu et al. 2011; Fischi-Gómez et al. 2015; Zhang et al. 2011; Schwingenschuh et al. 2010; Wen et al. 2011). The edge values were entered into a matrix, whose elements were fiber density values. We adopted a simple network model that considered undirected and weighted edges. The matrix was referred to as the connectivity matrix in this study.

**Table 2:** Structural connectivity values for the three groups

Connections	Structural connectivity (mean ± SD)			Corrected p value		
	NC	SWEDD	PD	PD-NC	SWEDD-NC	SWEDD-PD
Associative cortex–caudate	0.671 ± 1.381	0.299 ± 0.453	0.232 ± 0.319	0.017	0.054	0.214
Associative cortex–putamen	3.452 ± 1.561	3.471 ± 1.517	3.734 ± 1.771	0.228	0.487	0.232
*Associative cortex–thalamus*	*0.833* *±* *0.488*	*0.650* *±* *0.367*	*1.173* *±* *0.750*	*0.009*	*0.032*	*<0.001*
Limbic cortex–caudate	2.197 ± 1.463	1.902 ± 1.310	0.710 ± 1.215	<0.001	0.162	<0.001
Limbic cortex–putamen	3.175 ± 1.272	3.271 ± 1.474	2.557 ± 1.147	0.013	0.387	0.008
Limbic cortex–thalamus	1.535 ± 0.810	1.379 ± 0.866	1.054 ± 0.889	0.005	0.194	0.048
Sensorimotor cortex–caudate	0.430 ± 0.993	0.170 ± 0.471	0.053 ± 0.076	<0.001	0.062	0.016
*Sensorimotor cortex–putamen*	*1.250* *±* *0.742*	*1.485* *±* *0.930*	*1.728* *±* *1.108*	*0.011*	*0.010*	*0.027*
Sensorimotor cortex–thalamus	0.964 ± 0.574	0.947 ± 0.537	1.019 ± 0.703	0.325	0.511	0.292
Pallidum–caudate	0.676 ± 0.644	0.838 ± 0.744	0.631 ± 0.825	0.374	0.145	0.112
*Pallidum–putamen*	*2.166* *±* *0.898*	*1.840* *±* *0.917*	*1.283* *±* *0.522*	*<0.001*	*0.045*	*<0.001*
Pallidum–thalamus	0.799 ± 0.519	0.926 ± 0.523	0.565 ± 0.452	0.007	0.143	<0.001
Putamen–thalamus	0.439 ± 0.437	0.477 ± 0.388	0.346 ± 0.298	0.132	0.295	0.044
Substantia nigra–putamen	0.004 ± 0.017	0.001 ± 0.004	0.004 ± 0.015	0.496	0.179	0.192
Substantia nigra–thalamus	0.001 ± 0.005	0.004 ± 0.012	0.009 ± 0.003	0.193	0.045	0.223
Substantia nigra–pallidum	0.096 ± 0.243	0.095 ± 0.363	0.160 ± 0.129	0.210	0.485	0.350

Structural connectivity values based on fiber density were reported for 16 connections within the CBGT circuit. The values were reported as the mean ± standard deviation (SD). Group-wise differences in NC-PD, SWEDD-NC and SWEDD-PD comparisons were reported using corrected p values. The italicised text shows identified connections with significant differences among the three comparisons

### Statistical tests

Group-wise differences among the SWEDD, NC and PD groups were assessed by exploring 16 connections, which was equivalent to investigating 16 elements in the connectivity matrix. For each group, the connectivity matrices of participants were stacked into three-dimensional matrices. Each group had a single three-dimensional matrix. Each element in the stacked connectivity matrix contained 40 observations. We performed non-parametric permutation tests for 16 connections of interests to identify group-wise differences among the NC, SWEDD and PD groups. A permutation test is a non-parametric approach that does not require the estimated parameter to follow a normal distribution and has been widely adopted in neuroimaging research (Smith et al. 2013). We performed the permutation tests by randomly assigning NC, SWEDD and PD patients 10,000 times. One permutation involved randomly assigning the first 40 cases to the NC group, the next 40 cases to the PD group and the remaining 40 cases to the SWEDD group. Differences in structural connectivity were considered significant if they did not belong to the 95 % of the null distribution derived from the permutation tests (p < 0.05, corrected) (Nichols and Holmes 2002; Bullmore et al. 1999).

### Correlation with clinical scores

Correlation analysis was performed to detect possible links between structural connectivity and clinical scores. We pooled connectivity matrices between the groups (i.e., NC, PD and SWEDD) into a single matrix and then calculated the Spearman correlations using the MDS-UPDRS scores for each element in the matrix. Multiple comparison issues were adjusted using Holm–Bonferroni correction which accounted for 16 pair-wise correlation analyses.

### Classification using identified connections

The three significant connections were fed into a support vector machine (SVM) classifier framework with a quadratic kernel to separate the NC, SWEDD and PD groups. The technical details of SVM are found in review articles (Vapnik 1999). We applied the leave-one-out cross-validation method to distinguish training and test data, due to the limited number of subjects available. For example, given 40 NC and 40 PD cases, we assigned 1 case as the test case and used the remaining 79 cases as the training data for the SVM classifier. The process was repeated 80 times, choosing a different test case each time. The SVM classifier seeks a decision boundary that can effectively separate samples near the decision boundary. Classifier accuracy, sensitivity and specificity were computed by comparing the classifier outcomes with the known ground truth using MATLAB. The entire procedure was performed for the NC-PD, NC-SWEDD and PD-SWEDD classifications.

## Results

### Structural connectivity differences

Structural connectivity results (i.e., fiber density values) for the three groups are reported in Table 2, which shows the mean and standard deviation (SD) for the fiber density values of 16 connections. The values were computed from both hemispheres. Overall, nine connections were significantly different between PD and NC. Of these connections, seven connections were smaller in PD compared with NC and two connections were smaller in NC compared with PD (p < 0.05, corrected). Four connections were significantly different between SWEDD and NC. Among these connections, two connections were smaller in NC and two connections were smaller in SWEDD (p < 0.05, corrected). Nine connections showed significant differences between PD and SWEDD. Among these connections, seven connections were smaller in PD and two connections were smaller in SWEDD (p < 0.05, corrected). PD patients showed significant connection differences compared with NC in associative cortex–caudate, associative cortex–thalamus, limbic cortex–caudate, limbic cortex–putamen, limbic cortex-thalamus, sensorimotor cortex–caudate, sensorimotor cortex–putamen, pallidum–putamen and pallidum–thalamus connections. SWEDD patients showed significant differences compared with NC in associative cortex–caudate, associative cortex–thalamus, sensorimotor cortex–putamen, pallidum–putamen and SN–thalamus connections. PD patients showed significant connection differences compared with SWEDD in associative cortex–thalamus, limbic cortex–caudate, limbic cortex–putamen, limbic cortex–thalamus, sensorimotor cortex–caudate, sensorimotor cortex–putamen, pallidum–putamen, pallidum–thalamus and putamen–thalamus connections. Associative cortex–thalamus, sensorimotor cortex–putamen and pallidum–putamen connections were commonly identified as significant in NC-PD, SWEDD-NC and SWEDD-PD comparisons, as shown in italic font in Table 2.

### Correlation between identified connections and clinical scores

Correlation analysis was performed to identify possible links between structural connectivity and clinical score (i.e., MDS-UPDRS) for all 16 connections, as shown in Table 3. The correlation analysis results of the three previously identified connections are shown below. The pallidum–putamen connection showed a significant negative correlation between structural connectivity and clinical score (r = −0.352, corrected p = 0.001). We observed significant positive correlations between structural connectivity and clinical score (r = 0.280, corrected p = 0.014) for the sensorimotor cortex–putamen connection. No significant correlation for the associative cortex–thalamus connection was found (r = 0.088, corrected p = 1). In summary, two connections out of three showed a significant correlation with MDS-UPDRS score.

**Table 3:** Correlation between the structural connectivity and MDS-UPDRS III score

Connections	Structural connectivity (mean ± SD)				Corr coef. (corrected p value)
	NC	SWEDD	PD	Total	
Associative cortex–caudate	0.671 ± 1.381	0.299 ± 0.453	0.232 ± 0.319	0.401 ± 0.874	−0.171 (0.61)
Associative cortex–putamen	3.452 ± 1.561	3.471 ± 1.517	3.734 ± 1.771	3.552 ± 1.612	0.034 (1)
*Associative cortex–thalamus*	*0.833* *±* *0.488*	*0.650* *±* *0.367*	*1.173* *±* *0.750*	*0.886* *±* *0.595*	*0.088 (1)*
Limbic cortex–caudate	2.197 ± 1.463	1.902 ± 1.310	0.710 ± 1.215	1.603 ± 1.471	−0.404 (<0.001)
Limbic cortex–putamen	3.175 ± 1.272	3.271 ± 1.474	2.557 ± 1.147	3.001 ± 0.132	−0.070 (1)
Limbic cortex–thalamus	1.535 ± 0.810	1.379 ± 0.866	1.054 ± 0.889	1.323 ± 0.872	−0.239 (0.104)
Sensorimotor cortex–caudate	0.430 ± 0.993	0.170 ± 0.471	0.053 ± 0.076	0.217 ± 0.638	−0.161 (0.711)
*Sensorimotor cortex–putamen*	*1.250* *±* *0.742*	*1.485* *±* *0.930*	*1.728* *±* *1.108*	*1.488* *±* *0.951*	*0.280 (0.014)*
Sensorimotor cortex–thalamus	0.964 ± 0.574	0.947 ± 0.537	1.019 ± 0.703	0.977 ± 0.605	0.076 (1)
Pallidum–caudate	0.676 ± 0.644	0.838 ± 0.744	0.631 ± 0.825	0.715 ± 0.741	−0.099 (1)
*Pallidum–putamen*	*2.166* *±* *0.898*	*1.840* *±* *0.917*	*1.283* *±* *0.522*	*1.766* *±* *0.873*	*−0.352 (0.001)*
Pallidum–thalamus	0.799 ± 0.519	0.926 ± 0.523	0.565 ± 0.452	0.763 ± 0.517	−0.183 (0.54)
Putamen–thalamus	0.439 ± 0.437	0.477 ± 0.388	0.346 ± 0.298	0.421 ± 0.380	−0.177 (0.572)
Substantia nigra–putamen	0.004 ± 0.017	0.001 ± 0.004	0.004 ± 0.015	0.003 ± 0.013	0.037 (1)
Substantia nigra–thalamus	0.001 ± 0.005	0.004 ± 0.012	0.009 ± 0.003	0.003 ± 0.009	0.103 (1)
Substantia nigra–pallidum	0.096 ± 0.243	0.095 ± 0.363	0.160 ± 0.129	0.117 ± 0.261	−0.017 (0.851)

Structural connectivity values based on fiber density were reported for 16 connections within the CBGT circuit. Correlation between the structural connectivity and MDS-UPDSR scores is reported as the Spearman correlation coefficient with the corrected p value in the rightmost column. The italicised text shows identified connections with significant group-wise differences in NC-PD, SWEDD-NC and SWEDD-PD comparisons as reported in Table 2

### Classifier performance

The SVM classifier using a quadratic kernel was applied to separate the NC, SWEDD and PD groups. Classifier performance in terms of accuracy, sensitivity and specificity are reported in Table 4, classifying the NC-PD, NC-SWEDD and PD-SWEDD cases. Overall, the classification results were generally good (i.e., mean sensitivity, specificity and accuracy were 70.83, 79.17 and 75.00 %, respectively).

**Table 4:** Classifier performance to separate the NC-PD, NC-SWEDD and PD-SWEDD classifications

Group	Sensitivity (%)	Specificity (%)	Accuracy (%)
NC versus PD	62.5	85	73.75
NC versus SWEDD	62.5	82.5	72.5
SWEDD versus PD	87.5	70	78.75

## Discussion

In this study, we identified pallidum–putamen, sensorimotor cortex–putamen and associative cortex–thalamus connections as connectivity profile unique to SWEDD using structural connectivity analyses. Moreover, pallidum–putamen and sensorimotor cortex–putamen connections were correlated with the MDS-UPDRS score. First, our results revealed decreased structural connectivity in pallidum–putamen connection in PD patients compared with NC and SWEDD. Neuroimaging studies have reported altered functional or structural connectivity in PD compared with NC subjects (Kim et al. 2013; Sharman et al. 2013; Wu et al. 2009; Yu et al. 2013; Zhang et al. 2015). One functional connectivity study showed a decreased connection in pallidum–putamen using rs-fMRI (Sharman et al. 2013). Others reported decreased levels of degree centrality, a graph network measure of local importance in the supplementary motor area and putamen using rs-fMRI in PD compared to NC subjects (Wu et al. 2009). Structural connectivity studies of PD patients showed marked reduction in connectivity in the nigrostriatal tract (connections among SN, STN, putamen and pallidum) using DTI (Zhang et al. 2015). Thus, our results were consistent with previous findings. Second, our results revealed decreased structural connectivity in sensorimotor–putamen connection in PD patients compared with NC and SWEDD. One functional connectivity study using rs-fMRI showed increased connection in putamen and supplementary motor area, a sub-region of sensorimotor cortex in PD patients (Yu et al. 2013). Another rs-fMRI study reported an increase in degree centrality in the parietal cortex of PD patients compared with NC subjects (Wu et al. 2009). One study using track-based spatial statistics analysis showed that bilateral motor-related tracts, such as the cortico-fugal pathway that connects the motor cortex and cerebral peduncle via the internal capsule, had higher mean diffusivity values in PD patients than in NC (Kim et al. 2013). Another study using track density reported that primary somatosensory cortices showed a significantly increased track density (Ziegler et al. 2014). Our results were consistent with previous findings. Third, our results revealed decreased structural connectivity in associative cortex–thalamus connection in PD patients compared with NC and SWEDD. One functional connectivity study using rs-fMRI reported an increase in degree centrality in the dorsolateral prefrontal cortex, a sub-region of the association cortex, in PD patients (Wu et al. 2009). A comparable study investigating associative cortex–thalamus connection using structural connectivity for PD was largely lacking. Thus, our results were partially consistent with previous findings.

The pallidum–putamen and sensorimotor cortex–putamen connections were significantly correlated with clinical scores, while associative cortex–thalamus connection was not correlated with clinical scores. The pallidum–putamen connection was reported as a key pathway for motor control within the CBGT circuit (Obeso et al. 2000). The sensorimotor cortex–putamen connection was related to motor control via glutamatergic projections in the cortico-striatal pathway. Pallidum–putamen and sensorimotor cortex–putamen connections were correlated with MDS-UPDRS scores, which might corroborate the existing research. The associative cortex–thalamus connection was reported as playing a secondary role in processing motor information and thus, might be less linked to MDS-UPDRS scores than the other two connections (Purves et al. 2008). Thus, we believe that pallidum–putamen and sensorimotor cortex–putamen connections might form a structural connectivity profile unique to SWEDD that could be a potential imaging biomarker for future movement disorder research.

Our study had several limitations. Our study was limited by small sample size. Only 40 SWEDD patients had both DTI, T1 and T2-weighted MRI data available in the database and thus we were limited to 40 SWEDD cases. The PD patients in our study have greater motor impairments then SWEDD patients and thus the connectivity difference between two groups could have come from either dopamine differences or degree of motor symptom severity. Further research controlling for effects of motor symptoms are needed. Another confounding factor is the lateralization of PD symptom onset (Stewart et al. 2009; Weintraub et al. 2005). The PPMI data did not consider unilateral PD symptom onset and thus the differences in connectivity could have come from variations in lateralized symptom onsets. We limited connectivity analysis to 16 known connections within the CBGT circuit, which did not cover the entire brain. We intended to focus on known connections first, thereby establishing a baseline for further research. We adopted DTI to assess fiber information, but DTI cannot distinguish between efferent and afferent connections and model complex fiber orientations; in addition, its limited voxel resolution only allows DTI to account for major fiber tracts. Use of high angular resolution diffusion imaging (HARDI) allows for complex modeling within a voxel, but HARDI requires longer scan times than DTI. In many cases, DTI is the practical option for assessing in vivo fiber information. Brain networks can be assessed not only using DTI, but also by other imaging modalities including fMRI. Multi-modal analysis of the brain network will allow incorporation of complementary information derived from different modalities to better quantify SWEDD characteristics. To date, ground truth regarding diagnosis of SWEDD is difficult to achieve. One study reported that some SWEDD patients converted to PD while others did not (Batla et al. 2014). A longitudinal follow-up exam would allow us to better assess validity of SWEDD diagnosis. Our study retrieved data from a research database, which lacked such follow-up data. We believe future research should consider such longitudinal data. There has been an update to the criteria of SWEDD and PD, which might need to be applied to PPMI data so that classification of SWEDD cases could be validated (Postuma et al. 2015). This is also left for future work.

## Conclusions

In this study, we adopted connectivity analysis based on fiber density to characterize SWEDD patients compared to NC and PD patients. Connectivity analysis within the GBCT circuit was applied to NC (n = 40), SWEDD (n = 40) and PD (n = 40) participants. Pallidum–putamen, sensorimotor cortex–putamen and associative cortex–thalamus connections were significant (corrected p < 0.05) and could separate SWEDD from NC and PD patients in terms of structural connectivity based on fiber density. In addition, two of those connections, pallidum–putamen and sensorimotor cortex–putamen, were correlated with the MDS-UPDRS score (r = −0.352, corrected p = 0.001 and r = 0.280, corrected p = 0.014, respectively). Significant connectivity results were fed into a SVM classifier. The mean performance of NC-PD, NC-SWEDD and PD-SWEDD classifications were 70.83 % (sensitivity), 79.17 % (specificity) and 75.00 % (accuracy). These results confirmed the connections separating SWEDD from PD patients and NC were important features and well correlated with well-established clinical scores. We believe these connections could potentially serve as a unique structural connectivity profile that distinguishes SWEDD from NC and PD patients.

## Abbreviations

PD: Parkinson’s disease
SWEDD: scans without evidence of dopaminergic deficit
SN: substantia nigra
CBGT: cortico-basal ganglia-thalamo-cortical
NC: normal control
DTI: diffusion tensor imaging
rs-fMRI: resting state functional MRI
STN: subthalamic nucleus
ROI: region of interest
PPMI: Parkinson’s Progression Markers Initiative
MDS-UPDRS: Movement Disorder Society-Sponsored Unified Parkinson’s Disease Rating Scale
FA: fractional anisotropy
FACT: the fiber assignment by continuous tracking
CD: connection density
CE: connection efficacy
SVM: support vector machine
HARDI: high angular resolution diffusion imaging
